# Molecular detection of methicillin heat-resistant *Staphylococcus aureus* strains in pasteurized camel milk in Saudi Arabia

**DOI:** 10.1042/BSR20193470

**Published:** 2020-04-17

**Authors:** Amani H. Aljahani, Khaloud M. Alarjani, Zeinab K. Hassan, Manal F. Elkhadragy, Elsayed A. Ismail, Abdulrahman H. Al-Masoud, Hany M. Yehia

**Affiliations:** 1 Department of Physical Sport Science, Nutrition and Food Science, Princess Nourah Bint Abdulrahman University, Saudi Arabia; 2 Department of Botany and Microbiology, College of Science, King Saud University, Saudi Arabia; 3 Cancer Biology Department, Virology and Immunolgy Unit, National Cancer Institute, Cairo University, Egypt; 4 Biology Department, Faculty of Science, Princess Nourah bint Abdulrahman University, Riyadh 11671, Saudi Arabia; 5 Zoology Department, Faculty of Science, Helwan University, Cairo 11790, Egypt; 6 Department of Dairy Science, Faculty of Agriculture, Benha University, Benha 13518, Egypt; 7 Food Science and Nutrition Department, College of Food and Agricultural Sciences, King Saud University, Riyadh, Saudi Arabia; 8 Food Science and Nutrition Department, Faculty of Home Economics, Helwan University, Cairo, Egypt

**Keywords:** D-value, mecA gene, methicillin-heat resistant Staphylococcus aureus (MHRSA), Pasteurized camel milk, polymerase chain reaction (PCR), SDS-PAGE

## Abstract

Antibiotic- and heat-resistant bacteria in camel milk is a potential public health problem. *Staphylococcus aureus* (*S. aureus*) is an opportunistic pathogen in humans, dairy cattle and camels. We characterized the phenotype and genotype of methicillin-resistant staphylococcal strains recovered from pasteurized and raw camel milk (as control) distributed in the retail markets of Saudi Arabia. Of the 100 samples assessed between March and May 2016, 20 *S. aureus* isolates were recovered from pasteurized milk, 10 of which were resistant to cefoxitin, and as such, were methicillin-resistant. However, raw camel milk did not contain methicillin-resistant *S. aureus* (MRSA). Antimicrobial susceptibility tests showed that the resistance ratio for other antibiotics was 60%. We performed a polymerase chain reaction (PCR) assay using primers for the methicillin-resistant gene *mec*A and nucleotide sequencing to detect and verify the methicillin-resistant strains. Basic local alignment search tool (BLAST) analysis of the gene sequences showed a 96–100% similarity between the resistant isolates and the *S. aureus* CS100 strain’s *mecA* gene. Ten of the methicillin-resistant isolates were heat-resistant and were stable at temperatures up to 85°C for 60 s, and three of these were resistant at 90°C for 60 or 90 s. The mean decimal reduction time (*D*_85_-value) was 111 s for the ten isolates. Sodium dodecyl sulfate (SDS)/polyacrylamide gel electrophoresis (PAGE) showed that there was no difference in the total protein profiles for the ten methicillin heat-resistant *S. aureus* (MHRSA) isolates and for *S. aureus* ATCC 29737. In conclusion, a relatively high percentage of the tested pasteurized camel milk samples contained *S. aureus* (20%) and MHRSA (10%).

## Introduction

The foodborne pathogen *Staphylococcus aureus* (*S. aureus*), belonging to the *Staphylococcus* genus, is an opportunistic, nosocomial bacteria that has been previously reported in community-associated (CA) outbreaks worldwide [1,2]. A number of *S. aureus* strains are resistant to multiple antibiotics, including methicillin. These methicillin-resistant *S. aureus* (MRSA) strains harbor the penicillin-binding protein (PBP) 2a (PBP2a)-encoding gene *mecA* and its analog *mecC*. Both genes confer resistance to methicillin and other beta (β)-lactam antibiotics, and thus their presence in food is a global health concern and a threat to public health [3]. The widespread distribution of these strains may be likely a result of the overuse of antibiotics in human medicine and veterinary medicine. Antibiotics have also been used to promote growth in agriculture, although this process has been banned globally. In European countries, where substantial antibiotic resistance research has been performed, this practice has been prohibited for more than a decade [4].

MRSA has been found in livestock and is transmissible between humans and animals [5]. The prevalence of MRSA in livestock has increased and results in contaminated food products. *S. aureus* is considered one of the most important infectious etiological agents for mastitis in dairy cattle [6,7]. A large number of Food and Drug Administration (FDA)-approved antibiotics are commercially available for the treatment of mastitis in dairy cattle. These are divided into two classes of antibiotics: β-lactams (e.g., penicillin, amoxicillin, cloxacillin, ceftiofur, hetacillinand cephapirin) and lincosamides (e.g., pirlimycin). While the antibiotic methicillin is not used for the treatment of mastitis, MRSA has been isolated from bovine milk. Song et al. [8] isolated MRSA from the milk of cattle suffering from mastitis. The presence of MRSA in raw milk is a major public health concern. Moreover, one of the sources for MRSA infection in animals may be the environment since these bacteria are able to survive for several months [9].

Many MRSA strains are responsible for nosocomial infections and have a significant impact on patients. The prevalence of MRSA may also be associated with poor food handling practices, resulting in bacterial contamination. The illegal transport of food by passengers on international flights is a potential causal factor since crossing geographic borders is a transmission route for many antibiotic-resistant strains, especially for enterotoxigenic bacteria such as MRSA. Contaminated food of animal origin may contribute to the incidence of CA-MRSA [10,11] or hospital-acquired (HA)-MRSA [12,13]. Pasteurization is a process by which milk is treated at high temperatures to destroy potentially harmful pathogens. Two common types of pasteurization techniques are used to pasteurize milk: the low-temperature long-time (LTLT) process, whereby milk is heated to 63°C for 30 min and the high-temperature short-time (HTST) process, whereby it is heated to 72°C for 15 s, followed by sudden cooling to 4°C or to ambient temperature. However, it is believed that the cooling process is to improve milk quality (reduce milk protein breakdown from heat) and to minimize (but not completely destroy) the residual bacterial population (i.e., bacteriostasis). Therefore, the presence of MRSA in pasteurized camel milk, which is resistant to the standard pasteurization treatments, is of great concern for public health [14]. Alternative HTST processes such as 88.3°C for 1 s, 90°C for 0.5 s, 93.8°C for 0.1 s, 96.2°C for 0.05 s or 100°C for 0.01 s, can be used for the treatment of such milk (FDA).

Animal husbandry uses antimicrobial agents to promote growth of the animals. However, as a consequence, it has also increased the selection pressure in favor of the expansion of the resistant bacterial populations in animals and animal products [15]. The detection and isolation of *Staphylococcus* species from poultry-processing plants, chicken carcasses, milk and dairy products are evidence suggesting that resistant microorganisms or their antibiotic-resistance genes may be transferred to humans via food, animals or the environment [16].

However, little information is available about the prevalence of MRSA in camel milk, especially for those strains that exhibit both antibiotic- and heat-resistance. The aims of the present study were to (1) isolate and identify methicillin heat-resistant *S. aureus* (MHRSA) from pasteurized camel milk samples collected from Riyadh markets in Saudi Arabia, (2) determine antibiotic resistance in these isolates and (3) compare the thermal death time (decimal reduction value (D-value)) for MRSA and MHRSA strains at temperatures higher than that used for pasteurization to explain the latter’s presence in pasteurized milk.

## Materials and methods

### Samples

MRSA isolates were obtained from 100 pasteurized camel milk samples collected between March 2017 and May 2017 from retail markets in the Saudi Arabian city of Riyadh. Raw camel milk samples were obtained from farms near Riyadh and were used as controls. All samples were transported directly to the Laboratory of Food Microbiology at the College of Food and Agriculture Sciences, King Saud University and refrigerated at 4°C. The samples were collected over five separate trips (tensamples in duplicates per trip).

### Bacterial isolation and identification

*S. aureus* ATCC 29737 was used as the reference strain. *S. aureus* isolates were isolated from pasteurized camel milk in accordance with the EN ISO 6888-1 (the International Organization for Standardization) standard procedure [17]. Samples were streaked on to Baird–Parker agar (Oxoid, U.K.) supplemented with an egg yolk-tellurite emulsion (Oxoid, U.K.) and the plates were incubated at 37°C for 48–72 h. Samples from raw camel milk were used as controls. Bacterial colonies were first characterized for catalase, Staphylase and coagulase activities using rabbit plasma (bioMerieux) and by blood hemolysis. Five presumptive *S. aureus* colonies from every sample were further characterized using the following tests.

### Phenotypic assessment of slime synthesis in *S. aureus* strains cultured on Congo Red agar

Slime production by the *Staphylococcus* isolates was quantitated based on the color of colonies cultured on the Congo Red agar. Bacterial samples were streaked on to the Congo Red agar composed of tryptose soybean (30 g/l), sucrose (36 g/l), agar powder (20 g/l) and Congo Red (0.8 g/l), and incubated for 24 h at 37°C under aerobic conditions. Slime produced by the *Staphylococcus* isolates was compared with *S. aureus* ATCC 29737 (a slime-producing strain considered as a positive control) and *S. epidermidis* ATCC 12228 (non-slime-producing negative control). Slime production results were interpreted as per the method described by Kouidhi et al. (2010) [18]. Strains producing intensive black, black or reddish-black colonies with a rough, dry or crystalline consistency were considered as normal slime producers. Whereas, those producing smooth red or Bordeaux red-colored colonies were classified as non-producers of slime.

### Analytical profile index STAPH-IDENT strip system

*S. aureus* isolates were identified biochemically using the Analytical profile index (API) Staph system (BioMérieux, Marcy l’Etoile, France) in accordance with the manufacturer’s instructions. The seven-digit profile number is converted into an identification by using the APILAB software, version 3.3.3.

### Antimicrobial susceptibility test

An antimicrobial susceptibility test was conducted on the *S. aureus* isolates. The tested bacteria were obtained from overnight cultures of single colonies inoculated into the brain-heart infusion broth (BHI, Oxoid, U.K). The bacterial suspension was applied to the surface of Mueller Hinton agar plates (Oxoid, U.K.) and then tested using the agar disk diffusion method [19]. A total of 25 antibiotic disks (Oxoid, U.K.), each containing one of the following antibiotics/antibiotic cocktails were tested against the bacteria: cefoxitin (FOX, 30 μg), streptomycin (S, 10 μg), colistin (at two different concentrations: CT, 10 μg; 25 μg), polymyxin B (PB, 300 U), cefadroxil (CFR, 30 μg), sulphamethoxazole (RL, 25 μg), nalidixic acid (NA, 30 μg), amikacin (AK, 30 μg), bacitracin (B, 10 μg), kanamycin (K, 30 μg), cephalothin (KF, 30 μg), tetracycline (TE, 30 μg), chloramphenicol (C, 30 μg), ciprofloxacin (Cip, 5 μg), linezolid (LZD, 30 μg), ticarcillin (TIC, 75 μg), nitrofurantoin (F, 300 μg), erythromycin (E, 15 μg), vancomycin (VA, 30 μg), ampicillin (AMP, 10 μg), amoxicillin/clavulanic acid (AMC, 30 μg), sulfamethoxazole trimethoprim (SXT, 25 μg), piperacillin (PRL, 75 μg)and amoxicillin (AML, 25 μg). The diameters for the zones of inhibition (mm) were interpreted based on the National Committee for Clinical Laboratory Standards (NCCLS)’s recommended criteria for *S. aureus* [19] and were classified as either sensitive (S), intermediate (I) or resistant (R) strains.

### Extraction of genomic DNA

Genomic DNA was extracted from the *Staphylococcus* isolates using the G-spin™ genomic DNA extraction kit (iNtRON Biotechnology, Seoul, Korea) as per manufacturer’s instructions.

### Detection of *S. aureus mecA* genes by polymerase chain reaction test

Total genomic DNA was isolated from *S. aureus* ATCC 29737 and the MHRSA isolates using an AxyPrep™ bacterial genomic DNA miniprep kit (Axygen Scientific, Inc., U.S.A.) in accordance with the manufacturer’s instructions. The *mecA* methicillin-resistant gene, housekeeping 16S rRNA, and the *S. aureus*-specific Sa442 DNA fragment were amplified using the following primer sets:

*mecA-*F (ACGTTCAATTTAATTTTGTT), *mecA-*R (GCGATTGTATTGCTATTATC); 16s-F (GAAAGCGTGGGGATCAAACA), 16s-R (TTGCGGGACTTAACCCAACA) and Sa442-F (GTCGGGTACACGATATTCTTCACG), Sa442-R (CTCTCGTATGACCAGCTTCGGTAC).

Polymerase chain reaction (PCR) was performed in a 50-µl volume composed of 1× FIREPol® Master Mix Ready to Load (12.5 mM Mg Cl_2;_ Solis BioDyne, Tartu, Estonia), 2 µl primer mix (50 pmol), 5 µl DNA template (50 µg/ml) and 33 µl of ultrapure water. DNA was amplified in a MULTEGENE thermal cycler (Labnet International, Inc. Edison, NJ) as follows: 95°C, 5 min; followed by 30 sequential cycles of 94°C for 1 min, 52°C for 1 min, 72°C for 1 min; a final elongation step at 72°C for 10 min was performed after the completion of the cycles. The amplified PCR products, along with a 1 Kb DNA ladder (GeneCraft), were separated on a 1.5% agarose gel (Sigma–Aldrich) containing ethidium bromide (0.5 mg/ml, ROTH) by electrophoresis (30 min at 100 V in 10× Tris-Borate-EDTA buffer; BIO Basic, Inc.) and visualized using a visual image analyzer software (Syngene).

### Identifying and genotyping MRSA isolates by *mecA* gene and direct DNA sequencing

Direct sequencing of the PCR products was performed to identify the *mecA* genotypes of *S. aureus* and their integration sites. The PCR products were purified and labeled using commercial kits according to the manufacturer’s instructions (AxyPrep™ PCR clean-up kit, Axygen®, NY, U.S.A.; BigDye Terminator v3.1 cycle sequencing kit, Applied Biosystems, CA, U.S.A.; BigDye X Terminator purification kit, Applied Biosystems, CA, U.S.A.) and as previously described by Al-Shabanah et al. [20]. The samples were sequenced by an automatic ABI 3500 genetic analyzer (Applied Biosystems, U.S.A.). The nucleotide sequences of 598 bp were identified by sequence alignment with the known sequences in the GenBank database using the Basic Local Alignment Search Tool (BLAST), provided by the National Cancer Institute, U.S.A. (http://blast.ncbi.nlm.nih.gov/Blast.cgi).

### Cell extract preparation

Cell extracts were prepared by a previously described method [21], with some modifications. A small aliquot (100 μl) of overnight presumptive isolates were inoculated into 10 ml of BHI medium (Oxoid, U.K) and grown to an optical density (OD) of 0.6–0.8 (3–4 h) at 620 nm wavelength. Protein extract was prepared from 250 mg of cells in 100 μl TES buffer (50 mM Tris/HCl, pH 8, 1 mM EDTA, 25% sucrose) supplemented with 20 μl lysozyme (50 mg/ml), 5 μl mutanolysin (5000 μ/ml) and 5–10 μl of 20% sodium dodecyl sulfate (SDS). The contents were stored at −20°C until required.

### Polyacrylamide gel electrophoresis

Protein extracts from the isolates were separated by mass according to the method described by Scarcelli et al. [22]. Electrophoresis was carried out on a 12% polyacrylamide running gel, in a Tris/EDTA glycine-sucrose buffer (0.025 M Tris, 0.19 M glycine buffer, pH 8.3; containing 100 μl of sucrose buffer: 50 mM Tris/HCl, pH 8; 40 mM EDTA, pH 8; 0.75 M sucrose). Fifty microliters of extracts (standard and bacterial isolates) were loaded on to the gel. Gels were stained with 0.25% Coomassie Brilliant Blue R-250 (Bio-Rad Marnesla Coquette, France) and following a destaining step was used until the protein bands were visualized. Whole-cell protein profiles of presumptive MHRSA isolates were compared with *S. aureus* ATCC 29737 under denaturing conditions using SDS.

### Determination of D-value

Solutions containing the MRSA isolates and *S. aureus* ATCC 29737 were prepared for thermal destruction or inactivation trials. Active cultures were obtained by inoculating single colonies of the *S. aureus* strains into BHI broth and allowing them to grow at 37°C for 24 h. Each active culture was diluted into BHI medium at 1:9 ratio. Since active cultures of *S. aureus* tend to clump, the cells were dispersed by vortexing at maximum speed for 1 min prior to dilution in the BHI medium. Before heat treatment, the number of cells in the inoculated medium ranged from 10^6^ to 10^7^ per ml. Duplicate tubes containing the diluted solutions were exposed to different temperatures (85°C for MSRA isolates; 90°C and 95°C for MSRHA isolates) for a set time (0–60 s with increments of 10 s per sample set). After each heat treatment, the sample was quickly cooled for 20 s by immersing in ice-water and then diluted 100- and 1000-fold. One milliliter from each diluted sample was cultured on BHI agar for 24–48 h at 37°C. To characterize the surviving bacteria, the number of colonies formed on the agar after 24 h were counted as colony-forming units per ml (CFUs/ml). To determine the D-value, ODs of the cultures in each tube with diluted samples were recorded after incubating at 37°C for 24 h. The D-values were determined from the linear section of the survivor plots using a linear regression analysis. D-values are reported in seconds (s) and are defined as the time required to achieve a log reduction in the bacterial population at the designated temperature.

## Results and discussion

### Phenotypic detection of MRSA

#### Staphylase test

**Figure 1:**
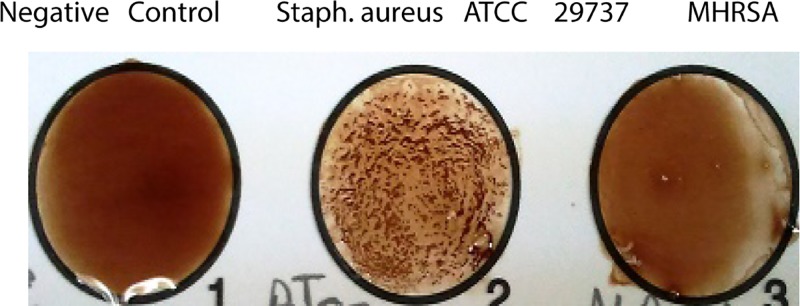
Staphylase test Red clumping (indicates the presence of clumping factor) clearly present with *S. aureus* ATCC29737 and absent for the negative control and MHRSA strain.

The Staphylase test is a rapid and accurate method to identify MRSA in laboratories, hospitals and food manufacturing industries, as the results can be observed within minutes. Staphylase kits detect agglutinating (clumping) factors and protein A present in *S. aureus* strains and are used routinely in the laboratory for the detection of *S. aureus*. As shown in Figure 1 and Table 1, *S. aureus* ATCC 29737 was positively identified by the Staphylase test (red clumps), whereas the ten MRSA isolates were negative for this test. This is consistent with previous reports showing that these assays are unable to identify MRSA [23–27]. Hsueh et al. [28] collected 25 MRSA isolates from December 1997 to March 1998, which exhibited negative Staphylase reactions.

**Table 1:** Phenotypic characterization and detection of MHRSA using a number of different laboratory tests

Microorganisms	Tests																											
	Staphylase	Growth on the Columbia Agar + 5% sheep blood	Growth on Mannitol Salt Agar	Growth on Mannitol Coagulase Agar	Growth on DNase Agar	Coagulase	Catalase	Glucose	Fructose	Mannose	Maltose	Lactose	Trehalose	Mannitol	Xylitol	Melibiose	Potassium nitrate	B-naphthyl phosphate	Sodium pyruvate	Raffinose	Xylose	Sucrose	Methyl α D-g glucopyranoside D- glucopyranoside	N-acetyl glucosamine	l-arginine	Urea	7-Digit numerical profile by API STAPH	Identification ratio (%)
								API STAPH SYSTEM TESTS																				
***S. aureus* ATCC 29737**	+	+	+	+	+	+	+	+	+	-	+	+	+	+	-	-	+	+	+	-	-	+	-	+	+	-	**6736153**	***S. aureus* (97%)**
**Isolate No. 1**	-	+	+	+	-	+	+	+	+	+	+	+	+	-	-	-	-	+	+	-	+	+	-	+	+	-	**6716151**	***S. aureus* (90%)**
**Isolate No. 2**	-	-	+	+	-	+	+	+	+	+	+	+	+	-	-	+	-	+	+	+	+	+	+	+	+	+	**6734151**	***S. aureus* (97%)**
**Isolate No. 3**	-	+	+	+	-	+	+	+	+	+	+	+	+	-	-	+	-	+	+	+	+	+	+	+	+	+	**6734151**	***S. aureus* (97.4%)**
**Isolate No. 4**	-	-	+	+	-	+	+	+	+	+	+	+	+	-	-	+	-	+	+	+	+	+	+	+	+	+	**6734151**	***S. aureus* (97.4%)**
**Isolate No. 5**	-	-	+	+	-	+	+	+	+	+	+	+	+	-	-	+	-	+	+	+	+	+	+	+	+	+	**6734151**	***S. aureus* (97.4%)**
**Isolate No. 6**	-	-	+	+	-	+	+	+	+	+	+	+	+	-	-	+	-	+	+	+	+	+	+	+	+	+	**6736153**	***S. aureus* (97%)**
**Isolate No. 7**	-	+	+	+	-	+	+	+	+	+	+	+	+	-	-	+	-	+	+	+	+	+	+	+	+	+	**6734151**	***S. aureus* (94.4%)**
**Isolate No. 8**	-	-	+	+	-	+	+	+	+	+	+	+	+	-	-	+	-	+	+	+	+	+	+	+	+	+	**6774151**	***S. aureus* (97.4%)**
**Isolate No. 9**	-	-	+	+	-	+	+	+	+	+	+	+	+	-	-	+	-	+	+	+	+	+	+	+	+	+	**6734151**	***S. aureus* (97.4%)**
**Isolate No. 10**	-	+	+	+	-	+	+	+	+	+	+	+	+	-	-	+	-	+	+	+	+	+	+	+	+	+	**6734151**	***S. aureus* (97.4%)**

#### Detection tests and API STAPH-IDENT system

Table 1 shows numerous tests for *S. aureus* by using rabbit plasma and in concordance with *S. aureus* ATCC 29737, the ten MRSA isolates were positive for coagulase activity. Although, MRSA isolates and methicillin-resistant coagulase-negative staphylococci isolates have been reported to be broadly resistant to penicillin and cephalosporin [29]. However, it also showed that while the reference strain was catalase-positive, the ten *S. aureus* isolates were also positive. While opposite with our results, Gruner et al. [30] isolated a catalase-negative MRSA that was identified by amplifying and sequencing the putative catalase gene. It was the first molecular description of a catalase-negative subspecies of the *S. aureus* strain. β-hemolysin, which is secreted by *S. aureus* breaks down red blood cells, causing a clear ring-like zone around the bacterial growth. Testing for β-hemolysis on sheep blood agar resulted in the appearance of clear zones around the *S. aureus* reference strain and four out of the ten MRSA isolates. However, a majority of the MRSA strains were negative for β-hemolysin. Therefore, β-hemolysis is another test that can be used to identify *S. aureus. S. aureus* reference strain and some of the MRSA isolates (1, 3, 7 and 10) were positive for β-hemolysin. However, other MRSA isolates (2, 4, 5, 6, 8 and 9) were found to be negative for β-hemolysin (Table 1).

Culturing bacteria on mannitol coagulase agar are to differentiate between different *Staphylococcus* species based on mannitol utilization and coagulase production. Mannitol is fermented when it is utilized. This is reflected by a change in the pH of the medium (purple to yellow), and appears as yellow zones surrounding coagulase-positive colonies. An opaque area composed of coagulated plasma also forms around the colonies of the coagulase-positive organisms such as *S. aureus*. Both the ATCC 29737 reference strain and all the MRSA isolates fermented mannitol and produced coagulase when cultured on mannitol salt agar, forming yellow-colored colonies surrounded by opaque yellow zones (Table 1).

Deoxyribonuclease (DNase) activity can also be used to distinguish among *Staphylococcus speci*es, as *S. aureus* is generally considered to exhibit a higher DNAse activity. As hypothesized, *S. aureus* ATCC 29737 was positive for DNase activity when cultured on DNase agar, which was visualized as clear zones around the colonies when the plates were flooded with hydrochloric acid. Unexpectedly, the MRSA isolates were negative in this assay. DiSalvo [31] confirmed a correlation between coagulase activity and DNase activity by incorporating DNA along with calcium chloride into the medium, activating the enzyme. However, our results did not support this relationship as, while all ten MRSAs were positive for coagulase activity, they did not exhibit DNAse activity (Table 1).

Many kits are commercially available for the identification of *S. aureus*, and different ones are used in different laboratories [32]. While the tube rabbit coagulase plasma test remains the gold standard for the identification of *S. aureus*, the time required to obtain precise results for this test is in the range of 4–24 h. In contrast, slide coagulase tests, including the tests with latex agglutination and hemagglutination methods that detect the presence of clumping factor or protein A, are more rapid and are attractive alternatives [33,34]. However, a major drawback for these commercial kits is their inability to accurately detect MRSA, where false-negative rates can be as high as 25% [35,36].

The existing literature does not conclusively differentiate between coagulase and clumping factor, the latter of which is a fibrinogen-binding protein on the bacterial cell surface. This lack of clarity stems from loose terminology, with the clumping factor sometimes referred to as the bound coagulase. Although coagulase is an extracellular protein, a small fraction is tightly bound to the bacterial cell surface where it can react with prothrombin. It has recently been shown that extracellular coagulases can bind fibrinogen as well as thrombin. Genetic studies showed unequivocally that coagulase and clumping factor are distinct entities. Specific mutants lacking coagulase retain the clumping factor activity, while clumping factor mutants express coagulase normally [37].

Results from the API STAPH-IDENT system test have been shown in Table 1, which revealed that all ten MRSA isolates presented seven-digit profile numbers that were consistent with an *S. aureus* identity*.* The percentage of identity for this relationship was between 90 and 97.4% according to the identification software (bioMérieux). Detection of MRSA by cefoxitin.

**Figure 2:**
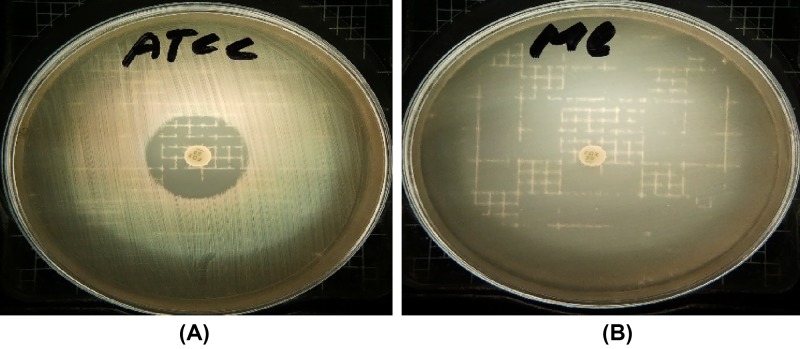
Antimicrobial resistance to cefoxitin (30 µg) (**A**) *S. aureus* methicillin-resistant isolates from camel milk (representative image) and in (**B**) *S. aureus* ATCC 29737 reference strain (positive control), which is methicillin sensitive.

Detection of the *mecA* gene or its product PBP2a is considered the gold standard for identifying MRSA species. However, not all laboratories are equipped to carry out molecular biology assays. Therefore, phenotypic methods such as antibiotic disk diffusion are used. The effect of cefoxitin (30 µg) on the growth of *S. aureus* ATCC 29737 and MRSA has been shown in Figure 2. As expected, *S. aureus* ATCC 29737 was sensitive to cefoxitin and exhibited a 25-mm inhibition zone diameter, whereas the growth of the MRSA isolates was not affected. Cefoxitin was used to identify MRSA strains because methicillin is no longer commercially available in the United States. Many researchers have used oxacillin as a substitute for methicillin. However, it has been found that cefoxitin is a stronger inducer of mecA. In comparison with oxacillin, cefoxitin has been shown to be less affected by penicillinase hyperproduction, which may imitate the methicillin-resistant phenotype [38–41]. Furthermore, cefoxitin has a high affinity for the staphylococcal PBP4, which, in combination with the overproduction of the PBP2, may also contribute to methicillin resistance [39]. Tests using cefoxitin have produced more reproducible and accurate results than tests using oxacillin [42].

According to the NCCLS disk diffusion criteria [43], a bacterial strain is considered resistant to cefoxitin when the zone diameter is ≤19 mm and susceptible when it is ≥20 mm. However, to identify methicillin resistance, Skov et al. [44] reported that in their tests using cefoxitin disks on the Oxoid IsoSensitest agar, a zone diameter of <29 mm was more appropriate to define resistance. They further suggest that this measurement may be a suitable guideline for both the disk diffusion and agar dilution methods. These tests clearly differentiate between MRSA and methicillin-susceptible *S. aureus* (MSSA) strains.

#### Slime production

**Figure 3:**
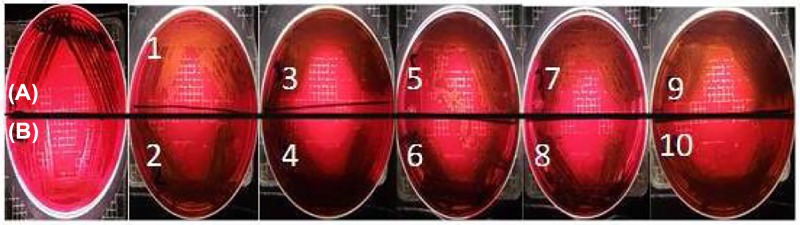
Slime production on Congo Red agar media (**A**) *S. aureus* ATCC 29737 (positive control: black colonies, upper sector), (**B**) *S. epidermidis* ATCC 12288 (negative control: pink colonies, lower sector) and (2–6): ten MHRSA isolates.

Slime production plays an important role for detection of the pathogenesis of infections caused by different microorganisms, especially staphylococci. It was detected in the *S. aureus* ATCC 29737 strain and also in all ten MRSA isolates (Figure 3). *S. epidermidis* did not produce slime. Many virulence factors that increase the potential for *S. aureus*-mediated diseases have been found to be secreted toxins that interfere directly with the host [45]. Slime production by *S. aureus* has been hypothesized to be a virulence factor. Our results were found to be in agreement with findings by other research groups showing that bacterial virulence was related to slime production in some *Staphylococcus* strains [46–50]. For example, the loosely bound exopolysaccharide layer (slime) of the capsule in coagulase-negative staphylococci (CNS) has been associated with sepsis, intravenous catheter-related bacteremia and other prosthetic device-associated infections [51–54]. Other strains of *S. aureus* with a bacterial capsule next to their bacterial cell wall may also have an extracapsular labile polysaccharide structure [55]. Some authors have reported that strains that produce slime had a greater capacity to colonize than those that do not. Hence, slime production by *S. aureus* strains may play a role in the establishment of infection [49,55]. It has also been reported that antibiotic resistance may be greater in slime-producing *S. aureus* strains than in their non-slime-producing counterparts [56].

#### Antibiotic resistance

All ten MRSA isolates exhibited resistance to 15 of the 25 tested antibiotics (60%). However, S. *aureus* ATCC 29737 was only resistant to 20% of these antibiotics (Table 2). Eight antibiotics were ineffective against MRSA isolates and *S. aureus* ATCC 29737. These were streptomycin, colistin (10 and 25 µg), polymyxin B, amikacin, sulphamethoxazole, bacitracin and kanamycin. Four antibiotics were highly effective against all bacterial strains tested. These included amoxicillin/clavulanic acid, sulphamethoxazole trimethoprim, piperacillin and amoxicillin.

**Table 2:** Antibiotic susceptibility test results of MHRSA and *S. aureus* ATCC 29737 24-h cultures using the antibiotic disk diffusion method

Strain number	Antibiotics																											
	FOX 30	S 101	CT 10	CT 25	PB 200	CFR 30	RL 25	NA 30	AK 30	B 10	K 30	KF 30	TE 30	CIP 5	LZD 30	C 30	TIC 75	F 300	E 15	VA 30	AMP 10	AMC 30	SXT 25	PRL 75	AML 25	Resistance	Intermediate	Sensitive
S □ I □ R □	≥20 to ≤19	≥22 to ≤14	ND	ND	ND	ND	ND	ND	≥17 15–16 ≤14	ND	≥18 14–17≤ 13	ND	≥19 15–18≤ 14	≥21 16–20≤ 15	≥21 to ≤20	≥18 13–17 ≤12	ND	≥17 15–16 ≤14	≥23 14 – 22 ≤13	≥15 - -	≥29 – ≤28	≥20 – ≤19	≥ 16 11–15 ≥ 10	ND	17≥ 14–16 ≥13	%	%	%
Strain 1	R	R	R	R	R	R	R	R	R	R	R	R	R	R	R	I	I	I	I	I	I	S	S	S	S	60	24	16
Strain 2	R	R	R	R	R	R	R	R	R	R	R	R	R	R	R	I	I	I	I	I	I	S	S	S	S	60	24	16
Strain 3	R	R	R	R	R	R	R	R	R	R	R	R	R	R	R	I	I	I	I	I	I	S	S	S	S	60	24	16
Strain 4	R	R	R	R	R	R	R	R	R	R	R	R	R	R	R	I	I	I	I	I	I	S	S	S	S	60	24	16
Strain 5	R	R	R	R	R	R	R	R	R	R	R	R	R	R	R	I	I	I	I	I	I	S	S	S	S	60	24	16
Strain 6	R	R	R	R	R	R	R	R	R	R	R	R	R	R	R	I	I	I	I	I	I	S	S	S	S	60	24	16
Strain 7	R	R	R	R	R	R	R	R	R	R	R	R	R	R	R	I	I	I	I	I	I	S	S	S	S	60	24	16
Strain 8	R	R	R	R	R	R	R	R	R	R	R	R	R	R	R	I	I	I	I	I	I	S	S	S	S	60	24	16
Strain 9	R	R	R	R	R	R	R	R	R	R	R	R	R	R	R	I	I	I	I	I	I	S	S	S	S	60	24	16
Strain 10	R	R	R	R	R	R	R	R	R	R	R	R	R	R	R	I	I	I	I	I	I	S	S	S	S	60	24	16
*S. aureus* ATCC 29737	S	R	R	R	R	S	R	I	R	R	R	S	S	S	S	S	I	I	I	S	I	S	S	S	S	20	20	48
Resistance%	90.9	100	100	100	100	90.9	100	100	100	90.9	90.9	90.9	90.9	-	-	-	-	-	-	-	-	-	-	-	-	-	-	-
Intermediate%	-	-		-	-	-	-	9.09	-	-	-	-	-	-	-	90.9	100	100	100	90.9	100	-	-	-	-	-	-	-
Sensitive%	9.09	-	-	-	-	9.09	-	-	-	-	-	9.09	9.09	9.09	9.09	9.09	-	-	-	9.09		100	100	100	100	-	-	-

Antibiotic sensitivity/resistance have been defined by the size of the inhibitory zone diameters as indicated for each antibiotics (mm). Mean zones of inhibition for common antibiotics tested: S, Sensitive; I, Intermediate; R, Resistant, except noted above and ND, not detected: treated as a common antibiotic inhibition zone. FOX, Cefoxitin (30 μg); S, Streptomycin (10 μg); CT, Colistin (10, 25 μg); PB, Polymyxin B (300 U); CFR, Cefadroxil (30 μg); RL, Sulphamethoxazole (25 μg); NA 30, Naldioxicacid (30 μg); AK, Amikacin (30 μg); B, Bacitracin (10 μg); K30, Kanamycin (30 μg); KF, Cephalothin (30 μg); TE30, Tetracyclin (30 μg); C 30, Chloramphenicol (30 μg); Cip 5, Ciprofloxacin (5 μg); LZD 30, Linezolid (30 μg); TIC 75, Ticarclline (75 μg); F300, Nitrofurantoin (300 μg); E 15, Erythromycin (15 μg); VA 30, Vancomycin (30 μg); AMP, Ampicillin (10 μg); AMC, Amoxy/clav.acid (30 μg); SXT 25, Sulphamethoxazole trimethoprim (25 μg); PRL, Piperacillin (75 μg); AML 25, Amoxicillin (25 μg).

The antibiotics that showed moderate effect were ticarcillin, nitrofurantoin, erythromycin and ampicillin. Therefore, the MRSA isolates were able to grow under stress conditions and resist against a broad spectrum of antibiotics. These characteristics may have arisen from exposure to a wide range of antibiotics during the feeding, watering and treatment of animals. People in economically developing areas who still consume raw milk without any thermal treatment have a greater risk of being exposed to more serious diseases.

MRSA strains can be divided into two categories: one carrying an innate resistance and the other carrying an acquired resistance. Innate resistance refers to an inherent lack of response to an antibiotic beyond its usual spectrum [57]. Acquired antibiotic resistance may develop in previously sensitive microorganisms, increasing the minimum inhibitory concentration (MIC) required for efficacy of a particular antibiotic [57]. High-grade acquired resistance occurs when there is a single-step mutation that develops during or after antibiotic therapy, resulting in an increased MIC that changes a previously susceptible isolate to one that is resistant to antibiotic levels beyond therapeutic doses [58]. Bacterial cell wall synthesis is inhibited by β-lactams, which act as suicide substrates for the transpeptidase domain of the PBPs [59]. Methicillin belongs to the β-lactam class of compounds that are hydrolyzed by β-lactamase. These include methicillin, cefoxitin, oxacillin, cloxacillin, dicloxacillin and nafcillin. Methicillin-resistant strains, in particular, methicillin-resistant coagulase-negative *Staphylococci*, have been found to be broadly resistant to penicillin and cephalosporins [29]. Methicillin resistance is most commonly mediated by *mecA*, which encodes for PBP2a and has a low affinity for all β-lactams [13]. Having a *mecA* gene is not sufficient for methicillin resistance since some *S. aureus* isolates that have the gene are susceptible to methicillin [60–62].

In a study that evaluated the efficacy of fosfomycin (FOM), which is used in combination with other antibiotics, it was found that FOM was less effective against MRSA. In a burn unit at the Tokyo Women’s Medical University Hospital, 12 different antibiotics were tested against 32 strains of MRSA isolated from the wounds of burn-injured patients. It was found that both ABK and VA were effective in suppressing *in vitro* bacterial growth, but the other antibiotics were ineffective as monotherapies. The combination of ABK or VA with FOM demonstrated efficacy against all the tested MRSA strains [63].

Upon the discovery of penicillin in 1944, the mortality rate of staphylococcal bacteremia decreased from ∼70 to ∼25% [62]. However, by the mid-1950s, mortality rates had returned to 45% as *S. aureus* became resistant to penicillin. Following the discovery of isoxazolyl penicillins such as methicillin and flucloxacillin, mortality rates from staphylococcal bacteremia again dropped to 25%. The development of methicillin resistance was documented almost as soon as these drugs became available [64]. Consequentially, mortality rates increased again. A meta-analysis of 30 studies revealed that the average mortality rate for MRSA was 36% in comparison with 24% for septicemia caused by MSSA [65]. Seven of the studies in this meta-analysis reported MRSA bacteremia mortality rates over 50%, with two that were over 80% [65,66]. Currently, the clinical consequences of resistance to VA continues to complicate the management of MRSA infections [67]. A mortality rate of 63% has been reported for patients who became infected with VA-intermediate *S. aureus* (VISA) [67]. The mortality was even higher (78%) in patients with septicemia caused by VISA.

### Genotypic detection of MRSA

#### Genomic DNA extraction

The amount of DNA extracted as quantified by fluorescence (Table 3) ranged from 53 to 81 µg/ml, and the quality of this DNA was examined by gel electrophoresis. Investigators using lysostaphin as a lytic agent during DNA extraction have found that lysostaphin resulted in an acceptable yield for PCR [68–70]. Johnson and Stell [71] reported that boiling the organisms in water followed by centrifugation to remove bacterial debris yields genomic bacterial DNA of sufficient quantity and quality to perform diagnostic PCR. Similarly, Buzinhani et al. [72] noted that DNA extracted using boiling water resulted in higher PCR sensitivity. It has been reported that genomic DNA yields ranging from 30 to 500 ng per 25 µl reaction are sufficient for PCR amplification, but yields below 30 ng have resulted in little or no detectable PCR product [73]. The same authors also found that a picogram amount of template DNA was sufficient for specific amplification when the annealing temperature was lowered, sometimes by as much as 10–12°C. However, this has the disadvantage of affecting the specificity, especially when diagnostic PCR is to be performed.

**Table 3:** Quantification of DNA extracted from *S. aureus* ATCC 29737 and the methicillin-resistant isolates (MRSA)

Isolate	DNA concentration µg/ml	Number of isolates	DNA concentration µg/ml
ATCC 29737 *S. aureus*	53	*S. aureus 2*	52
Isolate 1	80	Isolate 6	60
Isolate 2	80	Isolate 7	62
Isolate 3	52	Isolate 8	53
Isolate 4	80	Isolate 9	53
Isolate 5	79	Isolate 10	81

#### Identification of *mecA* in the *S. aureus* isolates

**Figure 4:**
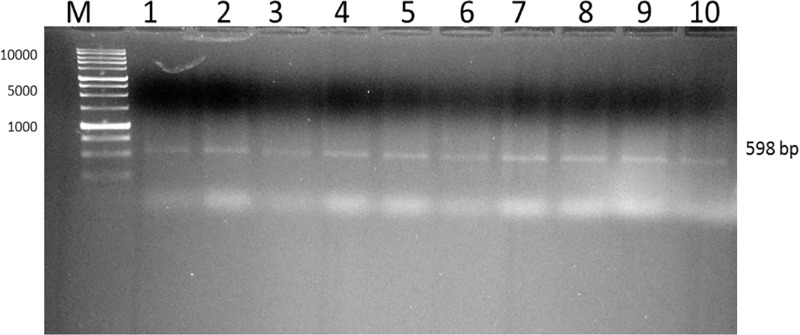
PCR product generated by using genomic DNA and *mecA* gene primer toxins Positive *mecA* bands appeared in the ten isolates (1–10) at 598 bp as visualized after gel electrophoresis using 1% agarose and 10 μl Ethidium Bromide with an image analyser (SYNGENE). M is DNA marker.

**Figure 5:**
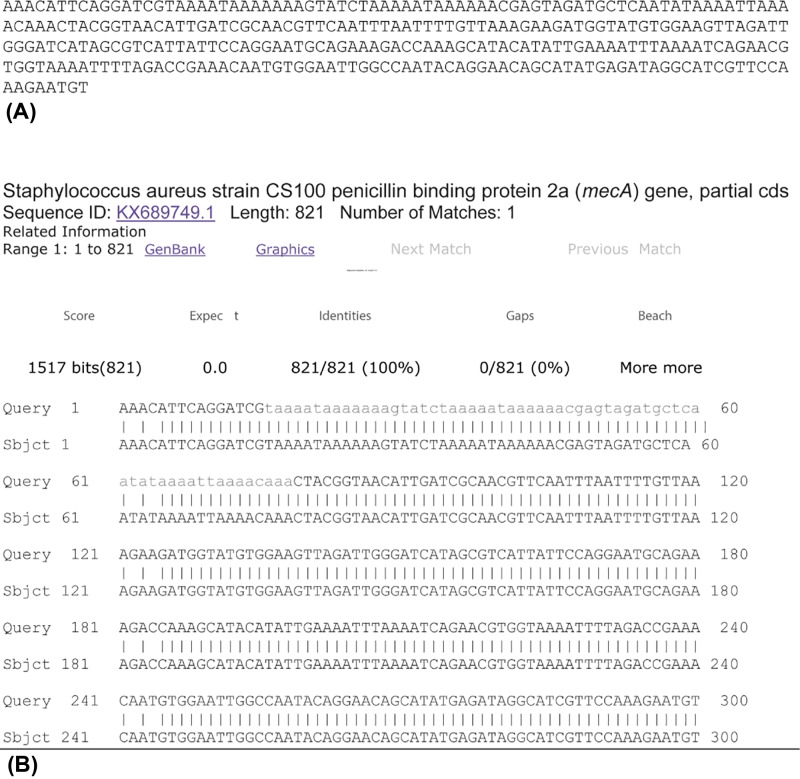
Partial nucleotide sequence of PCR product and summary of the BLAST result (**A**) A 300-bp segment of the PCR product from *S. aureus* using the *mecA* forward primer. (**B**) BLAST alignment of the sequence described in (A) with that of the *S. aureus* strain CS 100 PBP2a (*mecA*) gene.

Direct DNA sequencing of the MRSA DNA extracts using the *mecA-F* primer as the forward sequencing primer obtained hypervariable regions unique to the *S. aureus* strain (Figure 4). Submitting this 300-base pair (bp) sequence to the GenBank for BLAST alignment analysis generated a report that located the sequence to a region of the *S. aureus* strain CS100 PBP2a (*mecA*) gene (partial coding sequences, cds; GenBank accession number: KX689749.1; Figure 5).

Therefore, the use of single genes or DNA fragment-specific and species-specific 442-bp chromosomal fragment and 16S rRNA are a popular choice for the identification of *S. aureus* in multiplex PCR. As shown in Table 4, all isolates contained both the 16S rRNA and the Sa442 DNA, whereas *S. aureus* MRSA also contained the *mecA* gene. Ali and Mahmoud [74] isolated five MRSA isolates containing the *mecA* gene from raw camel milk distributed in the Egyptian town of Shalateen. Twenty-six isolates were found to be MSSA. The *mecA* gene and Sa442 DNA are popular DNA targets used for the rapid identification of MRSA. The Sa442 DNA fragment is an undefined 442-bp sequence that was originally described by Martineau et al*.* in 1998 [75]. Since then, there have been very few reports on *S. aureus* isolates that do not have an Sa442 fragment.

**Table 4:** PCR of the 16S rRNA, *mecA* and Sa442 genes in *S. auerus* ATCC 29737 and MRSA

Organisms	16 S rRNA		*MecA*		Sa442		Number of isolates
	PCR^+^	PCR^−^	PCR^+^	PCR^−^	PCR^+^	PCR^−^	
*S. aureus* ATCC 29737	1	-	-	-	1	-	1
*S. aureus* (MHRSA)	10	2	10	2	10	2	12

#### Total protein profile

**Figure 6:**
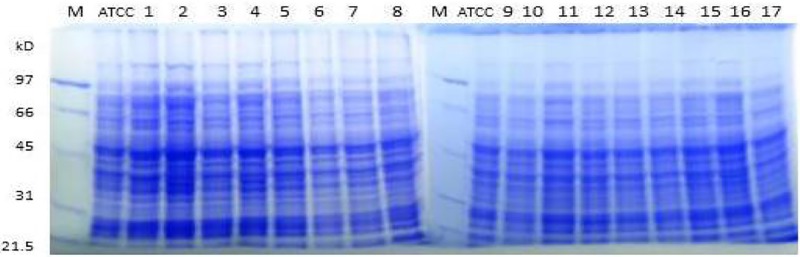
Total protein profiles of MRSA isolates by SDS/PAGE Lane M: the molecular weight standard. Lane ATCC: *S. aureus* total protein (positive control). Lanes 1–16: different MRSA isolates.

The total protein profile of the MRSA isolates were analyzed by SDS/polyacrylamide gel electrophoresis (PAGE). Protein was loaded and ran on a 12% SDS/PAGE gel. Reproducible profiles were obtained after staining with Coomassie Blue, as shown in Figure 6. The whole-protein profile of each isolate was similar in banding pattern and intensity in comparison with the reference *S. aureus* ATCC 29737 strain. However, due to a high degree of similarity among the samples, banding patterns were almost 100% comparable. This led to the conclusion that SDS/PAGE was not an effective tool to distinguish between the MRSA isolates. Therefore, all the isolates were closely related. These results were in agreement with those reported by Stephenson et al. [76].

#### Determination of D-values

**Figure 7:**
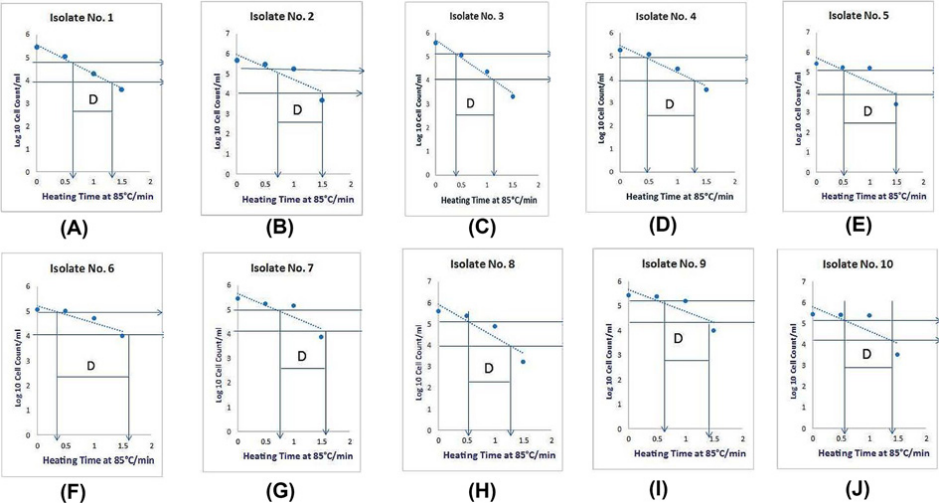
Thermal inactivation of ten *S. aureus mecA* isolates *S. aureus* exposed to 85°C for 0, 0.5, 1, 1.5 and 2 min. The D-values for each have been indicated by the letter D. D-value for the ten isolates of *S. aureus* (*mec* A) at 85 °C for 0, 0.5, 1, 1.5, 2 min (**A**-**J**).

**Figure 8:**
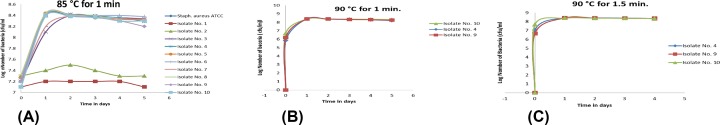
The effect of heat treatment on the viability count (CFU/ml) *S. aureus* ATCC 29737 and MHRSA isolates ten isolates of MHRS exposed to 85°C for 1 min (**A**), 90°C for 1 min (**B**) and 90°C for 1.5 min (**C**).

The D-value is the time required to inactivate or reduce 90% of the initial population, from 10^6^ to 10^7^ CFU/ml, at a given temperature. This time varies considerably among different strains of microorganisms but may be similar between those of the same genus and species, and is dependent on the treatment given to the microorganisms and the particular food contaminated with bacteria. As shown in Figure 7, the D-values for the ten isolates of *S. aureus mecA*, when treated at 85°C, was 108 s, 120 s, 120 s, 126 s, 138 s, 150 s, 120 s, 108 s, 120 s and 126 s, respectively. The mean *D_85_* value was 111 s. Figure 8A shows the results from heat treatment at 85°C for 60 s, with all isolates cultured in a liquid medium. The *S. aureus* ATCC 29737 strain and isolates numbers 3, 6 and 7 failed to grow. Isolates 1 and 2 grew poorly in BHI, whereas isolates 4, 5, 8, 9 and 10 grew well. When the treatment temperature was elevated to 90°C for 60 and 90 s, no growth was observed for three of the remaining isolates (4, 9 and 10; Figure 8B,C). No isolates grew at the 95°C for 120 s condition. Some isolates of MRSA that were viable at high temperatures (ranging from 85 to 90°C) were considered to be heat-resistant strains and thus were called MHRSA.

Liquid or solid nutrient-rich media such as BHI was more conducive to the growth of injured *S. aureus* cells than other complex media or defined media such as the Baird–Parker agar, which lead to a delay in growth. Similarly, milk contains more nutritious components that favor the growth of *S. aureus* as the dominant microbe following pasteurization. *S. aureus* ATCC 29737 did not grow at 85 or 90°C. Kennedy et al. [77] determined the D-values of *S. aureus* under pre-chilled and un-chilled storage conditions and found that the *D_50_, D_55_* and *D_60_* values ranged from 2658 to 2674 s, 780 to 1302 s and 288 to 390 s, respectively.

## Conclusions

Our research showed that pasteurized camel milk contained a high proportion of MHRSA (10%). This prevalence was high and contributes to a genuine risk to public health. The presence of this microbe in camel milk is a potential problem that should be referred to the pasteurized camel-milk industry since it lowers the quality and safety of this product for their consumers. The appearance of heat-resistant strains may have resulted from the use of high temperatures, beyond those used during pasteurization, giving rise to new proteins that were resistant to elevated temperatures. Therefore, we suggest that different temperatures (and times) may be used to process these kinds of milk, for example, 93.8°C for 0.1 s, 96.2°C for 0.05 s or 100°C for 0.01 s, in order to destroy contaminating MHRSA.

## Abbreviations

API: analytical profile index
BLAST: basic local alignment search tool
CA: community-associated
CFU/ml: colony-forming unit per ml
DNase: deoxyribonuclease
D-value: decimal reduction value
FDA: Food and Drug Administration
FOM: fosfomycin
HTST: high-temperature short-time
MHRSA: methicillin heat-resistant *S. aureus*
MIC: minimum inhibitory concentration
MRSA: methicillin-resistant *S. aureus*
MSSA: methicillin-susceptible *S. aureus*
NCCLS: National Committee for Clinical Laboratory Standard
OD: optical density
PAGE: polyacrylamide gel electrophoresis
PBP: penicillin-binding protein
PCR: polymerase chain reaction
SDS: sodium dodecyl sulfate
*S. aureus*: *Staphylococcus aureus*
VISA: vancomycin-intermediate *S. aureus*
